# Multimodal Investigation into the Interaction of Quinacrine with Microcavity-Supported Lipid Bilayers

**DOI:** 10.1021/acs.langmuir.2c00524

**Published:** 2022-05-13

**Authors:** Nirod Kumar Sarangi, Amrutha Prabhakaran, Tia E. Keyes

**Affiliations:** School of Chemical Science and National Centre for Sensor Research, Dublin City University, Dublin 9, Ireland

## Abstract

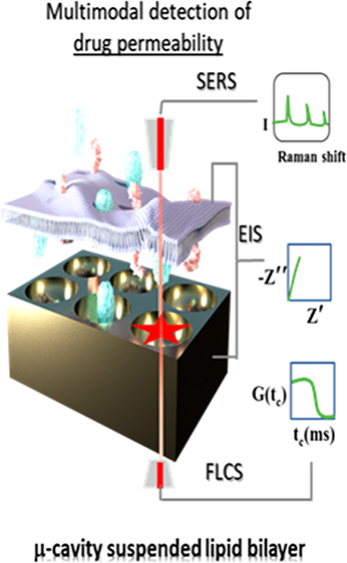

Quinacrine is a versatile drug that is widely recognized for its antimalarial action through its inhibition of the phospholipase enzyme. It also has antianthelmintic and antiprotozoan activities and is a strong DNA binder that may be used to combat multidrug resistance in cancer. Despite extensive cell-based studies, a detailed understanding of quinacrine’s influence on the cell membrane, including permeability, binding, and rearrangement at the molecular level, is lacking. Herein, we apply microcavity-suspended lipid bilayers (MSLBs) as *in vitro* models of the cell membrane comprising DOPC, DOPC:Chol(3:1), and DOPC:SM:Chol(2:2:1) to investigate the influence of cholesterol and intrinsic phase heterogeneity induced by mixed-lipid composition on the membrane interactions of quinacrine. Using electrochemical impedance spectroscopy (EIS) and surface-enhanced Raman spectroscopy (SERS) as label-free surface-sensitive techniques, we have studied quinacrine interaction and permeability across the different MSLBs. Our EIS data reveal that the drug is permeable through ternary DOPC:SM:Chol and DOPC-only bilayer compositions. In contrast, the binary cholesterol/DOPC membrane arrested permeation, yet the drug binds or intercalates at this membrane as reflected by an increase in membrane impedance. SERS supported the EIS data, which was utilized to gain structural insights into the drug–membrane interaction. Our SERS data also provides a simple but powerful label-free assessment of drug permeation because a significant SERS enhancement of the drug’s Raman signature was observed only if the drug accessed the plasmonic interior of the pore cavity passing through the membrane. Fluorescent lifetime correlation spectroscopy (FLCS) provides further biophysical insight, revealing that quinacrine binding increases the lipid diffusivity of DOPC and the ternary membrane while remarkably decreasing the lipid diffusivity of the DOPC:Chol membrane. Overall, because of its adaptability to multimodal approaches, the MSLB platform provides rich and detailed insights into drug–membrane interactions, making it a powerful tool for *in vitro* drug screening.

## Introduction

**Figure 1:**
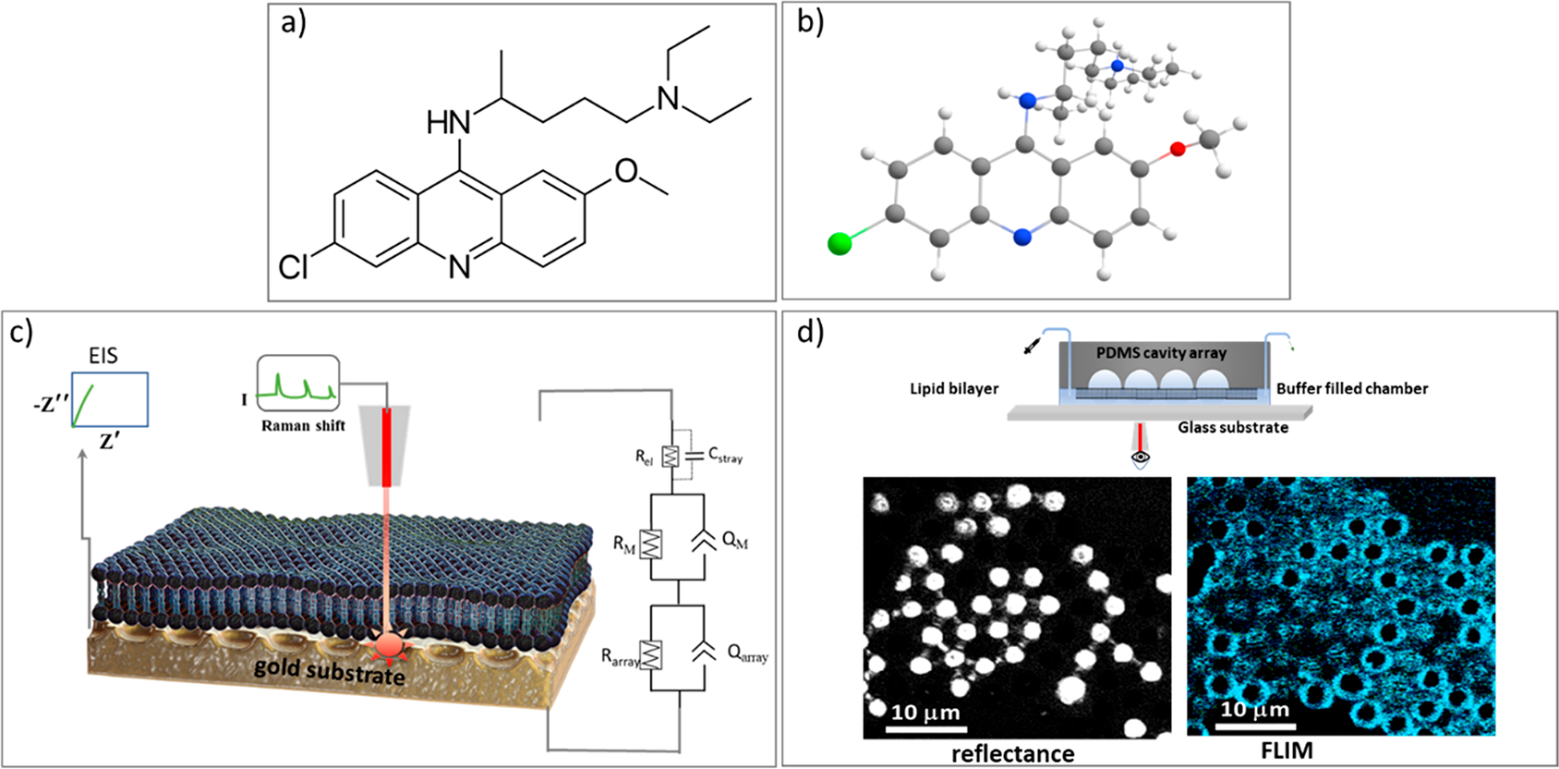
(a) Chemical structure and (b) DFT (CAM-B3LYP)-optimized structure of quinacrine (grey, carbon; white, hydrogen; green, chlorine; red, oxygen; and blue, nitrogen). For clarity, double bonds are not displayed. (c) Schematic representation of a gold-microcavity-suspended lipid bilayer (MSLB) array used in this work (not to scale) for dual detection using the EIS and SERS methods. On the right is shown the equivalent circuit model (ECM) used herein to fit the EIS data. In the ECM, *R*_el_ and *C*_stray_ represent the solution electrolyte resistance and stray capacitance, *R*_M_ and *Q*_M_ represent the membrane resistance and constant phase element (CPE), respectively, and *R*_array_ and *Q*_array_ are the microcavity array resistance and CPE. (d) Schematic representation of the PDMS-microcavity-based microfluidic device (top) for fluorescent lifetime imaging and FCS study. The bright round shape (bottom left) shown in the reflectance image corresponds to the aqueous-filled cavities and (bottom right) shows the corresponding fluorescence lifetime image in the same area obtained from DOPC MSLB doped with 0.01 mol % of fluorescently labeled lipid probe DOPE-ATTO655. The dark regions in the reflectance image are the cavities that buffer did not fill, and bright ring-like structures in the FLIM image are unfilled cavities where bilayer failed to span. This distinction meant it was facile to ensure that filled/cavity suspended bilayers were always selected for study. It is important to note that the bilayer forms a continuous film on gold due to smaller pore sizes and surface chemistry.

Quinacrine (Figure 1a) is a drug with multiple therapeutic activities. Used as an antimalarial drug for nearly a century, it is currently under investigation as a cancer chemotherapeutic.^1^ Its antimalarial efficacy is due to its action as a β-hematin inhibitor, which results in increased levels of free hemin, triggering oxidative stress via peroxidase reactions, eventually stopping proteolysis, and causing damage to the parasite’s membrane.^2,3^ Quinacrine has more recently shown significant promise as an anticancer agent.^4,5^ It is a potent Ca^2+^ channel blocker,^6^ it inhibits tumorigenesis in endometrial cancer (EC),^7^ and it induces p53, a tumor-suppressor protein, while inhibiting NFκB signaling, resulting in antitumor activity.^8^ Furthermore, recent studies on quinacrine have shown that it binds to and intercalates with DNA.^1,9^ It has thus been repurposed as a chemotherapeutic drug, primarily for gynecological malignancies^4^ and lung cancer,^10^ and is currently undergoing a phase 2 clinical trial in the treatment of prostate, lung, and colorectal cancer.

Quinacrine is lipophilic and thus likely to interact strongly with the plasma membrane. In a parallel artificial membrane permeation assay (PAMPA), quinacrine was shown to be permeable to the blood–brain barrier (BBB) lipid composition.^11,12^ Although limited in terms of the structural mimicry of the biological membrane, such assays have been shown to provide a fair correlation with cell permeability.^13,14^

It has been reported that antimalarial drugs including quinacrine, which is encapsulated within dendritic micelles, are delivered to *Plasmodium*-infected red blood cells (pRBCs).^15^ In the absence of specific receptor binding sites, studies have shown that quinacrine has limited interaction with the zwitterionic phosphocholine (PC) lipid membrane but binds avidly to acidic phosphatidylglycerol (PG) phospholipids.^16^ A concerted method in which quinacrine binds first to the phospholipid membrane (mainly phosphatidylglycerol, PG) and then intercalates into the membrane, inhibiting the activity of phospholipase A2 (PLA2),^16^ has been hypothesized. Goodman et al. reported evidence of the direct interaction of quinacrine with erythrocyte and platelet membrane phospholipids.^17^ In addition, the binding between lipid and quinacrine has reportedly been found to block the *Torpedo* nicotinic acetylcholine receptor at the lipid–protein interface.^18,19^

Despite quinacrine’s therapeutic versatility and a variety of intriguing pharmacokinetic properties, details of its interactions with the lipid bilayer and the role of membrane physicochemical properties such as membrane fluidity and the presence of phase-separated heterogeneous domains mimicking mammalian cells have not been studied to date for this drug. A molecular-level understanding of the drug–membrane interaction and drug permeability through membranes in a realistic biomimetic lipid bilayer model may provide crucial insights into the potential passive permeability for drug screening before moving on to more expensive cell-based studies. In this regard, we investigate herein the role of membrane composition in quinacrine–membrane binding and permeability utilizing surface-sensitive methods on a microcavity-supported lipid bilayer. Our goal is not only to show how different membrane compositions impact drug binding and permeability but also to demonstrate the potential of a microcavity-supported lipid membrane platform as a physiologically mimetic device for *in vitro* drug–membrane interaction testing.

So far, the parallel artificial membrane permeability assay (PAMPA)^20^ and the immobilized artificial membrane (IAM)^21^ have been two of the most frequent and relatively easy *in vitro* approaches for the passive mode of small-molecule drug permeability across the cell membrane test. Although both have proved to be reliable, their biomimicry is limited because neither represents a true bilayer with a hydrophobic core and reflects the actual thickness and asymmetry of the plasma membrane bilayer. A microcavity-supported lipid bilayer may offer a useful, low-cost alternative for studying drug interactions with the membrane during the early stages of drug development.^22^ Previously, *in vitro* platforms were used to anticipate passive membrane permeability and membrane-associated toxicological problems isolated from the complexity of the living cell.^23,24^ Biomembrane models, such as liposomes and supported lipid bilayers (SLB), have been successfully applied to interrogate the interaction of membrane lipids with small molecules.^25−30^ They have provided much insight, but there is still room for improvement in the biomimicry of the *in vivo* membrane. In the case of liposomes, they are limited to interfacial studies in two dimensions, while interference from the interfacial support due to pinning on the fluidity and functionality of the bilayer and associated proteins limits SLB biomimicry.^31−34^ Several modifications have been developed that may decouple the proximal leaflet from the substrate while retaining membrane component mobility. These include tethered lipid bilayer membranes and cushioned bilayer membranes.^35−40^ The advantage of SLB-based approaches is that they are more amenable to experimental interrogation than liposomes.^41−45^ In particular, when the solid support is a conducting metal electrode, it can be used for both electrochemical and vibrational spectroscopic studies as a label-free approach, providing diverse insights into the biophysical aspect of drug–membrane interaction at the molecular level.

Alternative approaches that build membranes supported over aqueous micropores to improve the fluidity without the use of tethers while maintaining stability have evolved. Most importantly, in the case of buffer-filled pore-suspended bilayers, they offer the advantage of a relatively deep aqueous reservoir in contact with the proximal leaflet, which SLBs do not have.^46−51^ We recently reported on such microcavity array-supported lipid bilayers (MSLBs) made from polystyrene sphere-templated gold and PDMS substrates, which we used to explore drug–membrane^52−55^ and protein–membrane^56−58^ interactions.

Herein, using multimodal detection methods, we investigate how quinacrine interacts with three distinct bilayer compositions: DOPC, DOPC:Chol(3:1), and DOPC:SM:Chol(2:2:1). The first two represent fluidic liquid disordered phases without and with cholesterol to investigate the influence of cholesterol on the permeability of such a phase and a ternary composition known to form both liquid disordered and ordered phases. Previously, it was shown that the heterogeneity caused by the long acyl chain of SM might improve drug permeability by encouraging polar contact.^59,60^ Thus, the membrane compositions that were explored were selected on the basis of their different membrane homogeneities and fluidities to understand how these factors influence the quinacrine interaction.^61−64^ We used electrochemical impedance spectroscopy (EIS) as a label-free and surface-sensitive technique that can detect drug binding, permeation, and thickness changes by measuring the membrane admittance. Fluorescence lifetime correlation spectroscopy (FLCS) was used to understand any drug-induced lipid mobility changes, and molecular structural insights on quinacrine–membrane interactions are obtained from surface-enhanced Raman spectroscopy by exploiting the plasmonic properties of the gold microcavity pore array.

## Materials and Methods

### Materials

1,2-Dioleyl-*sn*-glycerophosphocholine (DOPC) and *N*-(octadecanoyl)-sphing-4-enine-1-phosphocholine (SM) in powder form were purchased from Avanti polar lipids (Instruchemie, The Netherlands). Cholesterol and quinacrine of high purity (>99%) were obtained from Sigma-Aldrich (Wicklow, Ireland) and were used as purchased. 1,2-Dioleoyl-*sn*-glycero-3-phosphoethanolamine-labeled ATTO655 (DOPE-ATTO655) was purchase from ATTO-TEC GmbH (Siegen, Germany). Poly(dimethylsiloxane) silicon elastomer (PDMS) was purchased from Dow Corning GmbH (Wiesbaden, Germany). Gold disk electrodes consisting of silicon wafers coated with a 100 nm layer of gold on a 50 Å layer of titanium adhesive were obtained from AMS Biotechnology Inc. Monodisperse polystyrene latex spheres were obtained from Bangs Laboratories Inc. The commercial cyanide-free gold plating solution (TG-25 RTU) was obtained from Technic Inc. All other HPLC-grade reagents were obtained from Sigma-Aldrich and used as obtained. Ultrapure water with a resistivity 18.2 MΩ cm was produced by a Milli-Q (Millipore Academic) system and used for buffer preparation.

### Methods

#### Fabrication of a Gold Microcavity Array

Gold microcavity array electrodes were prepared by polystyrene (PS) microsphere lithographic techniques as reported previously.^52,54,56,65^ In brief, 100 nm gold-coated silicon wafer electrodes (Platypus Technologies, USA) of ∼1 cm× 1.1 cm were cut and cleaned by washing with THF followed by ethanol and then drying under a gentle stream of HP N_2_. The electrodes were then plasma treated for 5 min to render the surface hydrophilic. After plasma treatment, 1% (v/v) PS spheres (1 μm diameter) were drop cast over the gold chip and allowed to spread uniformly across the whole area. The chip was left overnight without shaking in order to evaporate the solvent to form a hexagonally close-packed self-assembled microsphere array. Next, the controlled electrochemical deposition of gold was carried out using a well-characterized amperometric *I*–*t* curve that can be used to determine precisely when gold is ∼0.5 μm thick^66^ using a three-electrode electrochemical workstation (Figure S1a, SI). After gold deposition, the substrates were rinsed with water and subsequently electrochemically cleaned using 0.05 M H_2_SO_4_ with cyclic voltammetry (Figure S1b, SI) in order to remove any oxide layers formed. After cleaning, the substrate was rinsed with Milli-Q water, dried under N_2_, and soaked for at least 48 h in an ethanolic solution of 1 mM 6-mercapto-1-hexanol (MH) to form a self-assembled monolayer (SAM). As described previously, the PS spheres were permitted to remain in place to act as a template to prevent the MH SAM from assembling in the pore interior, limiting the SAM to the interstitial planar regions at the top surface of the array between pores.^42,56^ The chemical modification of the substrate in the MH treatment step improves the stability of the bilayer by promoting the wettability of the gold substrate with an −OH-functionalized interface. After SAM formation, the substrates were washed with ethanol to remove any unbound thiol and then soaked in THF for 5 min. This step results in the complete removal of PS spheres leaving periodic micropore arrays with pore diameters of 1 and 0.5 μm depth with SAM adsorbed at the interstitial planar regions. After microcavity array fabrication, the cavities were washed with ethanol 2 or 3 times and subsequently rinsed 8–10 times with Milli-Q water, and the substrate was submerged in PBS buffer for at least 24 h before its use in MSLB preparation.

#### Fabrication of a PDMS Microcavity Array

The arrays were prepared in PDMS for optical studies. Mica sheets a few micrometers thick were cut to ∼1 × 1 cm^2^ dimensions and glued to glass cover slides. To form a PS array over the mica surface, ∼50 μL of ethanolic solution of 0.1% 4.61 μm PS was drop cast onto the flat mica surface. After ethanol evaporation, PDMS was poured onto the PS assembled at the mica sheet and cured at 90 °C for 1 h. After cooling, the PDMS was removed gently to reveal the PS array within a thin chamber of identical dimensions provided by the mica sheet thickness, which was suitable for confocal imaging and spectroscopy.^52,67^ The microcavity array was then formed by dissolving the PS sphere template from the PDMS substrate in tetrahydrofuran (THF) for 15 min via sonication. The substrates were then left to dry overnight. The resulting PDMS cavity arrays were hexagonally packed, and the pore diameter, determined by atomic force microscopy, was ∼2 μm (Figure S1c, SI). The PDMS substrates were plasma cleaned using oxygen plasma for 5 min to make the surface hydrophilic, and the microcavities were filled with buffer by sonication and stored inside buffer for further use.

#### Preparation of Microcavity-Spanned Bilayer Membranes

A combination of Langmuir–Blodgett (LB) and vesicle fusion (VF) methods was employed respectively for the assembly of the proximal and distal leaflets of lipid bilayers over buffer-filled gold and PDMS microcavity arrays according to a slight modification of a previously reported method.^42,67^ In brief, for Langmuir–Blodgett transfer, either single or multicomponent lipid mixtures were first dissolved in chloroform (∼1 mg/mL), and then ∼50 μL of lipid solutions was added to the KN2006 Langmuir trough (KSV-NIMA technology). A lag time of 10 min was set before compressing and decompressing (four cycles) the monolayers below the collapse surface pressure, and then the monolayers were held at a constant surface pressure of 33 mN/m for 5 min. The submerged gold/PDMS cavity array substrates in the LB trough were withdrawn from the water surface vertically at a speed of 5 mm/min to form the proximal leaflet of the bilayer. For vesicle preparation, the lipid solution (1 mg/mL) was dried in a 1.5 mL glass vial under a gentle stream of N_2_ gas to form a thin film. The dried lipid films were rehydrated in 1 mL of 0.01 M phosphate buffer saline (PBS) at pH 7.4 and vortex mixed for a period of 30–60 s. Next, the lipid suspensions were extruded 11 times through a 100 nm polycarbonate filter using a miniextruder (Avanti Polar Lipids) to form large unilamellar vesicles (LUV) that were then diluted to a final concentration of 0.25 mg/mL. The monolayer-modified cavity array was then submerged in the vesicle solutions for 1 h to allow them to fuse and form the MSLB. Next, the bilayer was gently washed with excess PBS buffer to remove any unfused vesicles, and the process was carried out in such a way that at no point during the process were the bilayers exposed to air.

#### Electrochemical Impedance Spectroscopy, EIS

The electrochemical measurements were performed with a CH760A potentiostat (CH Instruments, USA). A standard three-electrode cell was composed of a gold microcavity suspended bilayer as the working electrode, an Ag/AgCl (1 M KCl) reference electrode, and a platinum wire auxiliary electrode. The EIS data were measured over a frequency range of 0.05 to 10^5^ Hz with an ac modulation amplitude of 0.01 V at a potential DC bias of 0 V (vs Ag/AgCl). All measurements were carried out in a glass cell (approximate volume of 4 mL) in contact with PBS buffer maintained at pH 7.4. The EIS of the aqueous filled microcavity array coated with the lipid bilayer was measured initially prior to the addition of drugs to ensure signal stability. The non-Faradaic EIS signal from the MSLBs was evaluated for stability, and it was found that when initially placed in contact with the PBS buffer at 0 V an initial fluctuation of resistance was seen that stabilized within an hour and then remained unchanged over a prolonged window (10–12 h). This 10 h window of stability is well beyond our experimental time window (3 to 4 h) for drug-binding studies. A time lag of 60–90 min was allowed for each membrane composition to ensure that it had equilibrated in the electrochemical cell (no EIS fluctuation) before drug solutions were titrated. The electrochemical impedance response of the lipid bilayer was then measured for each drug concentration (0–20 μM). Each measurement took approximately 4 min, and was carried out at room temperature (22 ± 1 °C). The measured data were analyzed using Z-View software with an equivalent circuit fitting model (ECM) (Figure 1c), as described earlier,^54,55^ to estimate the membrane resistivity and capacitance values before and after drug interaction. The circuit consists of a parallel combination of solution resistance (*R*_s_) and a capacitor in series with a parallel combination of a constant phase element, CPE (*C*_array_), and cavity resistive elements (*R*_array_) of the microcavity array, and the membrane is approximated by a resistive element (*R*_M_) in parallel with a CPE (*Q*_M_). A constant phase element (CPE) is used in the equivalent circuit instead of pure capacitors because the impedance of solid electrodes usually deviates from that of the pure capacitor as a result of microscopic chemical inhomogeneity on both the electrode surface and the lipid bilayer. Depending on the composition, as described below, from EIS the bilayer resistance for an intact bilayer ranges from 2 to 10 MΩ (compared to the kΩ resistance of the SAM-modified cavity array prior to bilayer deposition). We previously showed that this resistance range corresponds to an intact SLB, so we used the resistance values to validate the bilayer prior to measurement.^52,55^ Poorly formed bilayers tended to have a lower resistance and were discarded.^55^

#### Surface-Enhanced Raman Spectroscopy (SERS) Measurements

The gold-microcavity-supported lipid bilayers in contact with PBS at pH 7.4 were studied with Raman spectroscopy using a confocal microscope (Horiba, U.K.) equipped with LabSpec software, LabSpec 5.45.09. 785 nm, and were used for excitation through a 100 μM pinhole equipped with a dispersion grating with 1200 grooves/mm. For both excitation and detection, a 50× (air, NA:0.75) objective was used. The spectra were collected across a 200–3400 cm^–1^ spectral range using 1% laser power (0.1 mW) to avoid plasmonic heating that can damage the bilayer. An exposure time of 4 s and accumulation for 6 s were used for spectral recording. The instrument was calibrated using a Si(100) wafer calibrated to its standard peak at 520.7 cm^–1^ and the Rayleigh line before the measurement of the sample. The spectra of bulk materials and the powder form of the lipid or drug (∼2 mg) were collected from a flat gold substrate at a high laser power of 100 mW versus 0.1 mW for SERS measurements on the MSLB platform.

#### Fluorescence Lifetime Correlation Spectroscopy (FLCS)

Fluorescence lifetime imaging (FLIM) and fluorescence lifetime correlation spectroscopy (FLCS) measurements were performed using a Microtime 200 system (PicoQuant GmBH, Germany) integrated with an FCS module, a dual SPD detection unit, time-correlated single photon counting (TCSPC), and inverted microscope model Olympus X1-71 with a Olympus UPlan SApo 60×/1.2 water-immersion objective. The FLIM and FLCS measurements were acquired from a PDMS microcavity array assembled into the microfluidic device, as shown in Figure 1d (top). A single-mode optical fiber guides the lasers to the main unit and provides a homogeneous Gaussian profile excitation beam. The lasers were pulsed at 20 MHz, corresponding to an interval of 50 ns. The emitted fluorescence was collected through the microscope objective, a 635rpc dichroic mirror blocked the backscattered light, and HQ670lp AHF/Chroma 640 nm filters were used to clean up the signal. A 50 μm pinhole was used. Fluorescence was detected using a single photon avalanche diode (SPAD) from MPD (PicoQuant). The TCSPC enabled the simultaneous assessment of the lifetime in the nanosecond range along with the time of diffusion in the millisecond range. Furthermore, TCSPC in lifetime mode has the ability to filter out any contribution from after-pulsing and suppress scattered light and parasitic signals from the background. Before the FCLS measurement, backscattered images of the substrate (images were taken using an OD3 density filter, with marked reflectance in Figure 1d (bottom left)) and fluorescence lifetime images were acquired to ensure the optimal positioning of focus at the buffer-filled cavities where the bilayers are spanned (marked FLIM in Figure 1d (bottom right)). FLCS analyzes the time-dependent fluctuations of the fluorescence intensity ∂*I*(*t*) recorded over 30 s and conforms to an autocorrelation function, *G*(*t*_c_) = ⟨∂*I*(*t*)⟩·⟨∂*I*(*t* + *t*_c_)⟩/⟨∂*I*(*t*)⟩^2^, where ⟨⟩ denotes the time average and ⟨∂*I*(*t*)⟩ and ⟨∂*I*(*t* + *t*_c_)⟩ are the fluorescence intensity fluctuations around the mean value at times *t* and *t* + *t*_c_, respectively, where *t*_c_ is the lag time. The FLCS autocorrelation data was fitted to a 2D diffusion model, eq 1,
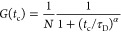
1where τ_D_ is the transit time, *N* is the average lipid probe number, and α is the anomaly coefficient. *G*(*t*_c_) is the autocorrelation measure of the self-similarity of a signal in time, i.e., the overlap of a signal with itself at various lag times *t*_c_. From the fitting, the τ_D_ values were evaluated and accordingly, and the diffusion coefficient can be obtained with eq 2
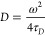
2where ω is the observation beam waist diameter typically obtained from the calibration of a standard dye diffusing in 3D with a known diffusion coefficient value.

## Results and Discussion

Many medications function intracellularly and thus must first cross the plasma membrane to reach their target. To better understand the drug–membrane interaction and its physicochemical significance in drug action, model lipid bilayers provide a convenient means to isolate the role of the membrane from the complexity of the cell. Biomimetic membranes that offer control over composition offer the opportunity to mimic particular aspects of cell membranes such as phase or lipid packing, and when combined with multiple interrogation methods, they can provide deep insight into how the drug and membrane interact. The present work unravels in detail the interaction of a well-known antimalarial drug, quinacrine, employing multimodal analytical approaches and a pore-supported lipid membrane platform. Using electrochemical impedance, surface-enhanced Raman spectroscopy, fluorescence lifetime imaging, and correlation spectroscopy, we examined the effects of quinacrine on three different membrane compositions.

### Studies of Quinacrine Interaction with a Membrane Using Electrochemical Impedance Spectroscopy (EIS)

**Figure 2:**
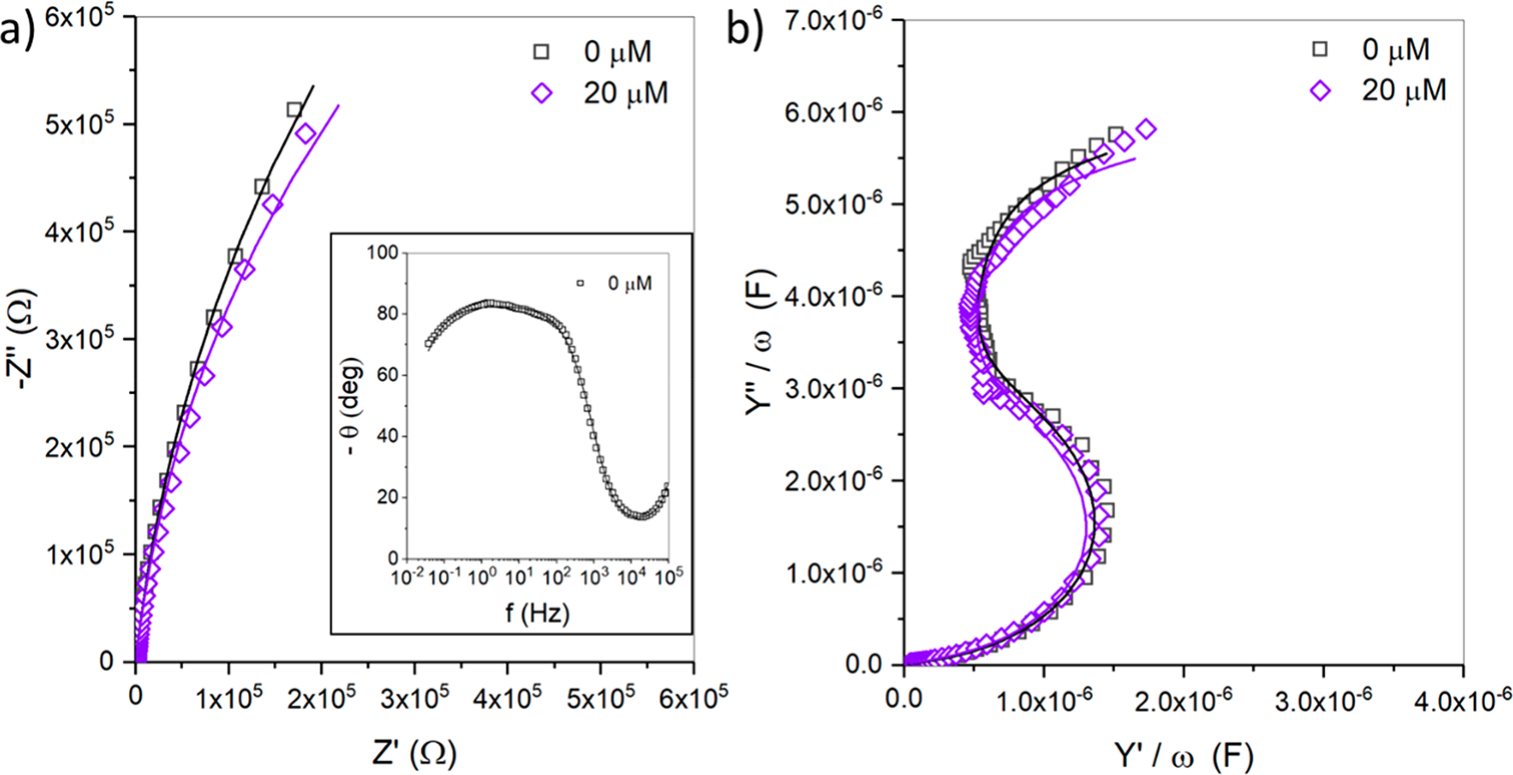
(a) Representative Nyquist plot and (b) frequency-normalized complex capacitance plot of the DOPC MSLB before (open black) and after 20 μM quinacrine (open violet) interaction, with their corresponding fit curves (solid lines) using ECM, as illustrated in Figure 1c. The Bode plot of the DOPC bilayer before drug addition is shown in the inset of (a). EIS measurements were performed in PBS buffer (pH 7.4) for frequencies ranging from 0.5 to 10^5^ Hz at 0 V DC bias potential (versus Ag/AgCl (1 M KCl)) with an AC modulation amplitude of 10 mV. All measurements were made at 22 ± 1 °C.

Electrodes composed of periodic pore arrays of 1 μm pore diameter and 0.5 μm depth, prepared as described previously,^52,54,56^ were used to assemble microcavity suspended lipid bilayers (MSLB) using a combination of the Langmuir–Blodgett and vesicle fusion methods. The interaction of quinacrine with pore-suspended bilayers made of a simple DOPC membrane was first studied using non-Faradaic electrochemical impedance spectroscopy in PBS buffer. Figure 2 depicts the representative non-Faradaic electrochemical impedance spectrum (EIS) responses for DOPC MSLBs before and after quinacrine (20 μM) addition to PBS. Changes to the EIS signal before (open black) and after the addition of a fixed concentration of quinacrine (open violet) can be represented by both the Nyquist (Figure 2a) and complex capacitance (Figure 2b) plots. The corresponding EIS data were fit to the previously described equivalent circuit model (ECM) (Figure 1c)^54,55^ to provide absolute membrane resistance and capacitance values. With this model, the changes in membrane resistance (*R*_M_) and capacitance (*Q*_M_) upon quinacrine addition are significant compared to those of other components in the ECM circuit. For example, the electrolyte solution resistance (∼35 ± 5 Ω), stray capacitance *C*_stray_ (∼1 nF) due to the electronics connector, cavity array resistance (*R*_array_ ≈ 1 kΩ), and capacitance (*Q*_array_ ≈ 40 μF s^*m*–1^, *m* = 0.5) remain essentially unchanged. Note that in the ECM model we employed a constant-phase element (CPE) rather than a pure capacitor because the impedance of gold porous electrodes deviates from that of flat gold owing to the presence of microscopic inhomogeneities in the electrode surface. The complex capacitance when a CPE is used can be expressed as 1/(*Qjω*)^*m*^, where *Q* is analogous to the magnitude of the capacitance, ω is the angular frequency (rad/s), and *m* is a homogeneity parameter varying between 0 and 1. *m* = 1 for an ideal capacitor, whereas *m* = 0 corresponds to a pure resistor. In our model, the *m* values for CPE at the membrane and array components are ∼0.93 ± 0.01 and 0.5 ± 0.02, respectively, which typically corresponds to the membrane when CPE approaches an ideal capacitor and the array CPE becomes a series RC circuit or Warburg impedance.^55^ Instead of a pure capacitor unit (F), the units of *Q*_M_ are F s^*m*–1^. Although the capacitance can be obtained from the *Q* value using the expression *C*(ω) = *Qω*^*m*–1^, it is valid only for a specific ω, limited to the specific ECM, and thus is not used in this study. Instead, as previously stated, we quote CPE(*Q*) as membrane capacitance values.

From Figure 2, the absolute resistance (*R*_M_) and capacitance (*Q*_M_) values of the pristine DOPC membrane (no drug) were found to be 2.58 ± 0.01 MΩ and 5.30 ± 0.02 μF s^*m*–1^ respectively. The addition of quinacrine (20 μM) reduces the *R*_M_ to 2.06 ± 0.02 MΩ and the capacitance (5.28 ± 0.02 μF s^*m*–1^) of the membrane. The aforementioned resistance and capacitance measurements have not been normalized to the active surface area. This is because during substrate fabrication the variation in PS sphere packing to 1 cm × 1.1 cm for flat gold electrodes causes inter-substrate variation in the electrode area, resulting in around a 2–5% fluctuation in the electrode area/roughness. Nonetheless, the active surface area of bare cavity array electrodes was ∼8–10 cm^2^ compared to that of flat gold electrodes (in the absence of a SAM and bilayer). Depending on the membrane composition and the total electroactive area of the gold electrodes explored in this work, the absolute values of membrane resistance and capacitance range from 20 to 80 MΩ cm^2^ and from 0.4 to 0.7 μF s^*m*–1^ cm^–2^, as calculated by multiplying and dividing by the electroactive area respectively. Similarily, because of small variations in the electroactive area from substrate to substrate due to variations in the fabrication method, such as pore packing, we report the relative changes in membrane resistance and capacitance rather than absolute values. Nevertheless, the absolute values of membrane resistance and capacitance obtained from this work are in agreement with previous reports for related biophysical models.^52,54,68−71^

**Figure 3:**
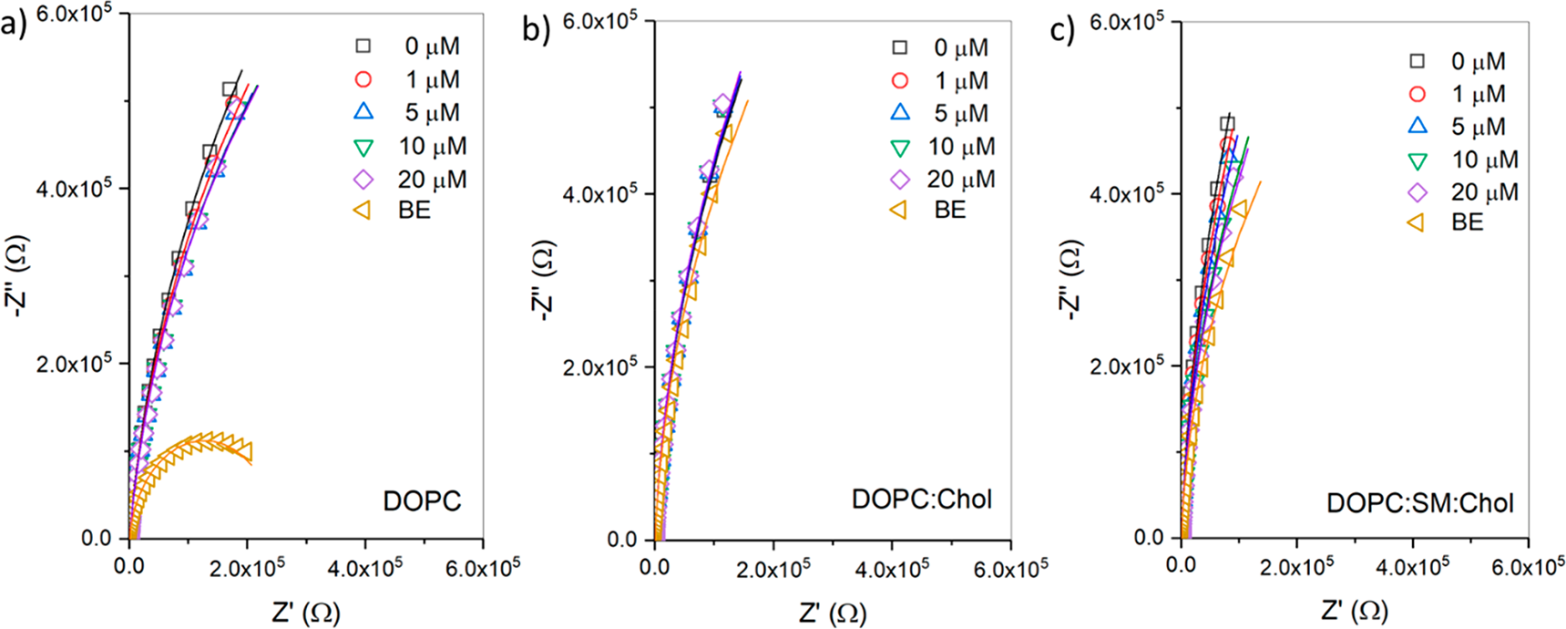
Non-Faradaic EIS data showing the Nyquist plots obtained from the titration of quinacrine in contacting solution at (a) DOPC, (b) DOPC:Chol (3:1), and (c) DOPC:SM:Chol (2:2:1) membranes suspended across gold microcavity arrays. The experiments were carried out in the three-electrode cell configuration, where the MSLB array on gold is the working electrode, Ag/AgCl (1 M KCl) is the reference electrode, and coiled Pt wire is the counter electrode. The electrolyte solution used was 0.01 M PBS solution (pH 7.4). EIS was recorded for frequencies between 0.05 and 10^5^ Hz at a DC bias of 0 V with an AC amplitude of 10 mV at 22 ± 1 °C. In each panel, the □, ○, Δ, ▽, and ◊ symbols represent 0, 1, 5, 10, and 20 μM quinacrine present in the contact solution, and ▷ represents the buffer exchange (BE) measurement (following postincubated sample to fresh PBS buffer). The measurements are performed in triplicate for each bilayer type.

Next, we measured changes in membrane impedance as the concentration of quinacrine in the contact solution changed from 1 to 20 μM (which is within the physiological concentration dose limit^6^) (Figure 3). Each drug concentration was added 10 min before measurement to allow for membrane incubation. To guarantee the membrane’s quality and stability, the EIS signal was monitored for 10–12 h prior to drug administration. After adding MSLB to the cell, resistance rises initially and equilibrates in 60–90 min depending on the membrane composition. The membrane impedance remained stable for 10 to 12 h after equilibration.^54,55^ The MSLB was rejected when the absolute resistance dropped below 1 MΩ and the capacitance exceeded 10 μF s^*m*–1^ during an hour of EIS operation. By 30 h, the resistivity had started to fall from the equilibrium value, establishing our stability window. Our drug titration experiments were all finished within 3 to 4 h, which is well within the window of stability. Prior to drug addition, the absolute membrane resistance values of DOPC, DOPC:Chol, and DOPC:SM:Chol were 2.58 ± 0.01, 2.82 ± 0.03, and 4.49 ± 0.02 MΩ, respectively, consistent with increased membrane ordering due to the tighter packing of alkyl chains of lipid tails in the presence of cholesterol and SM/Chol. The corresponding capacitance values were respectively 5.30 ± 0.02, 4.93 ± 0.22, and 4.42 ± 0.32 μF s^*m*–1^. Given the inverse relationship between the capacitance and thickness of the dielectric (in this case, the lipid bilayer), the result reflects an increase in membrane complexity as the membrane thickness increases.

The Nyquist plot (Figure 3) shows that the drug-induced response varies with membrane type. For example, with increasing quinacrine concentration in pure DOPC, the EIS signal shifts toward the real *Z*′ axis (Figure 3a), indicating that the drug reduces the bilayer impedance. The impedance of the ternary membrane (DOPC:SM:Chol) similarly decreases following drug interaction (Figure 3c); however, the decline is much greater than for DOPC. Increased ion permeation or membrane leakiness is typically linked to reduced lipid bilayer impedance. In contrast, for the DOPC:Chol membrane (Figure 3b), a modest but systematic increase in impedance is detected that only at the very highest drug concentration exceeds the experimental error.

To investigate the recovery of the membrane after drug interaction, we exchanged the drug-containing buffer for blank buffer, and all membranes showed decreased impedance. However, whereas DOPC exhibited a very large impedance decline (orange symbols in each panel of Figure 3), a much weaker decline was observed for DOPC:SM:Chol. Consistent with the tighter packing of this membrane and in spite of the apparent weak impact of the drug on membrane impedance, a measurable impedance decrease was also observed for the drug with DOPC:Chol on blank buffer exchange. EIS signals were monitored for 30 min after the buffer exchange, but the impedance did not recover for the DOPC membrane, indicating that the medication caused irreversible changes to the DOPC membrane.

In the absence of a bilayer, i.e., for SAM-only modified cavity array electrodes, adding 10 μM quinacrine to the cell elicited a small increase in resistance (*R*_array_) and a decrease in capacitance (*Q*_array_), possibly implying quinacrine adsorption in the interior of the cavity (Figure S2, SI). These alterations occur within 10 min of drug addition to the cell and remain unchanged after 6 to 7 h. We also observed a decrease in capacitance (*Q*_array_) for DOPC and DOPC:SM:Chol MSLB of comparable magnitude but not for DOPC:Chol, indicating quinacrine penetration across the former two membranes followed by a rise in the membrane CPE (*Q*_M_).

We extracted the absolute membrane resistance and capacitance values before and after drug interaction using the aforementioned ECM, where in order to get the relative changes (Δ), we normalized to the pristine membrane’s impedance. The relative resistance change (Δ*R*) is defined as *R*_M_^0^ – *R*_M_^drug^, where *R*_M_^0^ and *R*_*M*_^drug^ are the pristine membrane’s resistance without and with the drug, respectively, and the relative change in capacitance (Δ*Q*) is *Q*_M_^0^ – *Q*_M_^drug^ These values are provided in Tables 1 and 2, respectively.

**Table 1:** Relative Change in Resistance Values of Different Membrane Compositions after Quinacrine Titration Derived from a Nonlinear Least-Squares Fit Using the ECM Model

	Δ*R* (MΩ)		
quinacrine (μM)	DOPC	DOPC:Chol	DOPC:SM:Chol
0	0.0	0.0	0.0
1	–0.302 ± 0.02	0.022 ± 0.01	–0.703 ± 0.09
5	–0.47 ± 0.01	0.064 ± 0.00	–1.249 ± 0.13
10	–0.5 ± 0.08	0.097 ± 0.00	–1.915 ± 0.12
20	–0.52 ± 0.09	0.099 ± 0.00	–2.06 ± 0.10
buffer exchange	–2.33 ± 0.18	–0.493 ± 0.02	–2.81 ± 0.15

**Table 2:** Relative Changes in Capacitance Values of Different Membrane Compositions after Quinacrine Titration Derived from a Nonlinear Least Squares Fit to the EIS Data

	Δ*Q* (μF s^*m*–1^)		
quinacrine (μM)	DOPC	DOPC:Chol	DOPC:SM:Chol
0	0.0	0.0	0.0
1	0.04 ± 0.01	–0.025 ± 0.01	0.215 ± 0.01
5	0.07 ± 0.02	–0.016 ± 0.02	0.267 ± 0.07
10	–0.05 ± 0.03	–0.023 ± 0.01	0.272 ± 0.05
20	–0.06 ± 0.04	–0.047 ± 0.01	0.328 ± 0.51
buffer exchange	0.13 ± 0.03	0.144 ± 0.018	0.693 ± 0.14

**Figure 4:**
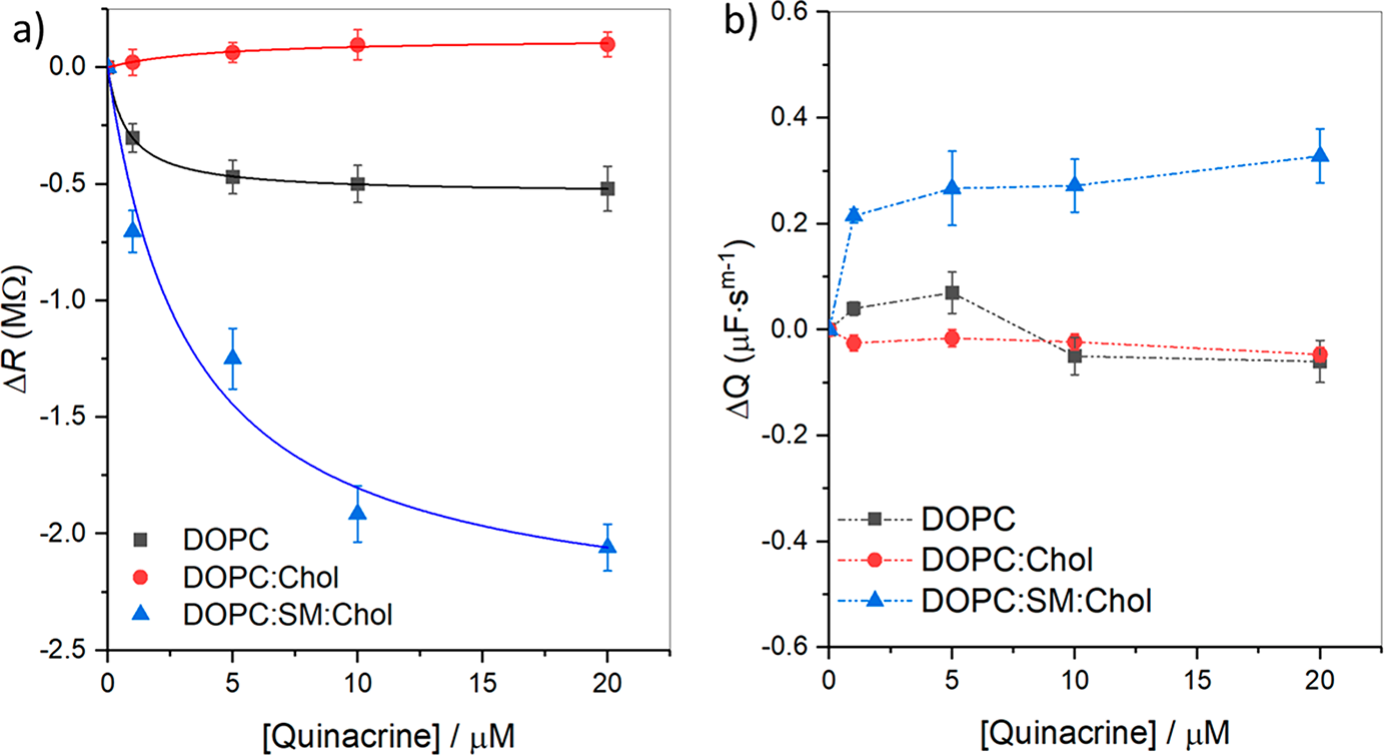
Relative change in membrane (a) resistance and (b) capacitance for a designated lipid composition versus quinacrine concentration. In each panel, the symbols ■, ●, and ▲ represent DOPC, DOPC:Chol and DOPC:SM:Chol membrane compositions. In panel (a), solid lines are the best-fit curves to the Langmuir isotherm model according to eq 3. Each data point given is a mean value ± SD and was assessed in triplicate for each bilayer type. The dashed lines in panel b are used to guide the eye.

The relative changes in resistance and capacitance values are plotted against quinacrine concentration and are displayed in Figure 4a,b, respectively. The Δ*R* versus quinacrine concentration data (filled symbols, Figure 4a) were fit (solid lines, Figure 4a) iteratively to the empirical Langmuir isotherm model described by eq 3, and the fit parameters of the Langmuir isotherm are provided in Table 3
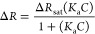
3where Δ*R* is the change in membrane resistance, Δ*R*_sat_ is absorption capacity or saturated binding of the drug, *K*_a_ is an empirical association constant, and *C* is the drug’s bulk concentration.^72,73^ As can be seen, as the drug concentration increases, the membrane resistance for DOPC and DOPC:SM:Chol membranes decreases. And as previously described, the ternary composition has the largest influence on membrane resistance, DOPC:SM:Chol (blue, Δ*R*_sat_ = −2.39 ± 0.24 MΩ) compared to DOPC (black, Δ*R*_sat_ = −0.536 ± 0.02 MΩ) (Figure 4a). Accordingly, the association constant, *K*_a_, values are found to be 0.30 ± 0.01 and 1.27 ± 0.02 L μM^–1^ for DOPC:SM:Chol and DOPC membrans, respectively. In contrast, DOPC:Chol (red) with a Δ*R*_sat_ = 0.126 ± 0.01 MΩ membrane showed a very small but non-negligible increase in relative resistance values. The *K*_a_ value for the DOPC:Chol bilayer was evaluated from the fit and found to be 0.23 ± 0.07 L μM^–1^.

**Table 3:** Data Obtained for the Quinacrine Drug by Fitting the Relative Change in the Resistance of Different Lipid Composition to a Nonlinear Langmuir Isotherm Model

lipid composition	Δ*R*_sat_ (MΩ)	*K*_a_ (L·μM^–1^)	*R*^2^
DOPC	–0.536 ± 0.01	1.27 ± 0.02	0.99
DOPC:Chol	0.126 ± 0.01	0.23 ± 0.07	0.98
DOPC:SM:Chol	–2.39 ± 0.24	0.30 ± 0.01	0.96

Figure 4b illustrates the relative change in capacitance (Δ*Q*) values versus quinacrine concentrations for the three different membrane types. Because we treat the membrane as a parallel plate capacitor, the capacitance is inversely related to the membrane thickness, and the Δ*Q* versus concentration plot can reflect alterations to the membrane thickness at different quinacrine concentrations. Although DOPC resistance data shows a large increase in membrane permittivity with increasing drug concentration, the membrane thickness remains relatively constant, reflected in only small changes in membrane capacitance (Figure 4b). Such decreased resistance without an accompanying change in capacitance may indicate pores/ion channels. It is worth noting that DOPC membrane thinning occurs at low drug concentrations (1 and 5 μM), but the capacitance then decreases at a higher quinacrine concentration attributed to drug accumulation at high drug concentrations (10 and 20 μM) (Figure 4b).

For DOPC:Chol capacitance, a negligible reduction in Δ*Q* occurred. Conversely, for DOPC:SM:Chol, Δ*Q* increases considerably in the presence of the drug, indicating membrane thinning. When the drug-containing solution is exchanged with fresh PBS buffer, EIS confirms that the membrane is still intact, as reflected by positive Δ*Q* values for DOPC:SM:Chol (0.693 ± 0.14 μF s^*m*–1^) (cf. Table 2).

### Surface-Enhanced Raman Spectroscopy of the Membrane–Quinacrine Interaction

**Figure 5:**
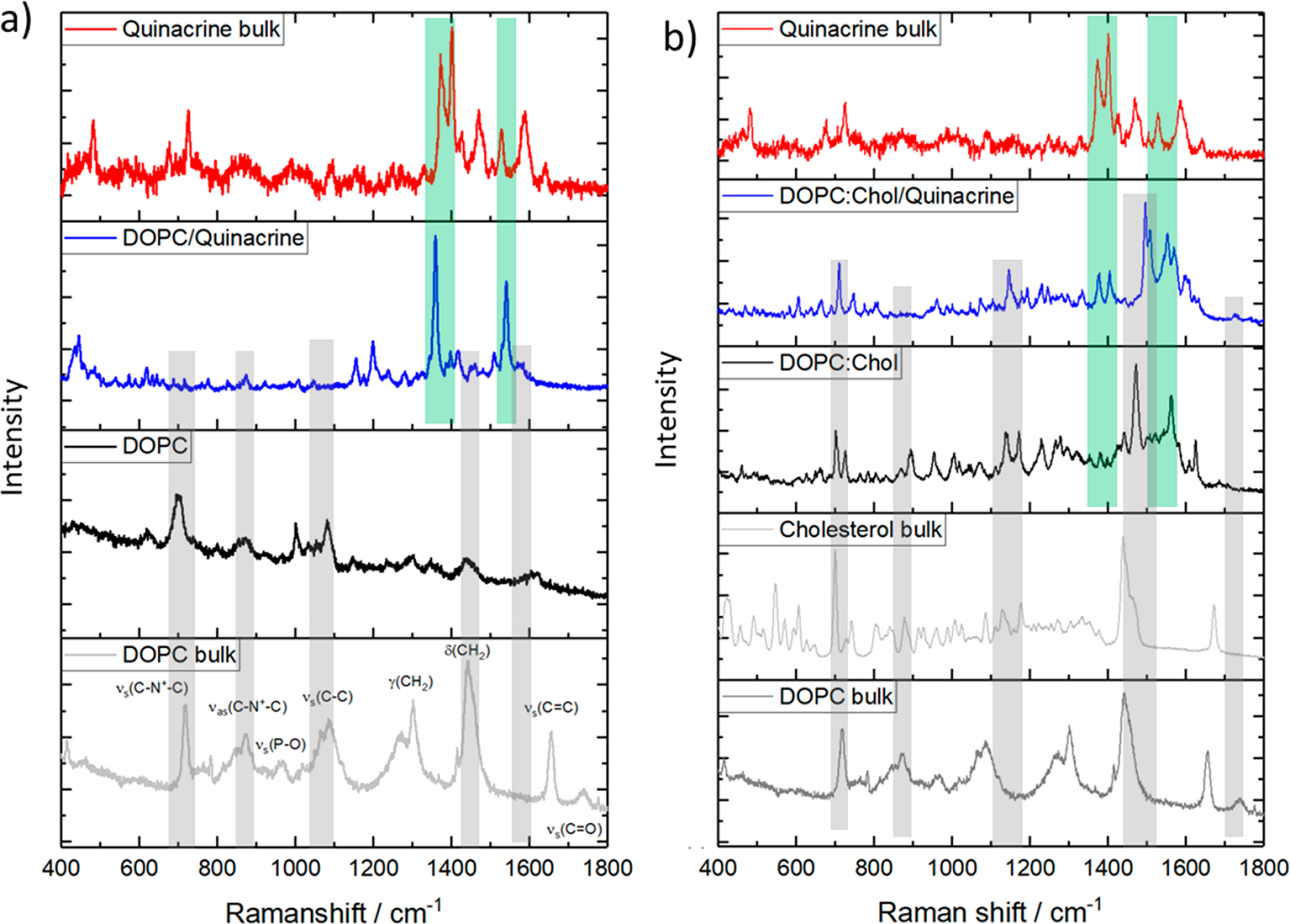
Surface-enhanced Raman spectroscopy (SERS) of (a) DOPC and (b) the DOPC:Chol (3:1) bilayer on a 1 μm cavity array without (black line) and with (blue line) the presence of quinacrine (10 uM). In each panel, classical Raman spectra of DOPC, cholesterol, and quinacrine in powder form (bulk) are displayed. The regions where the SERS band position matches the conventional lipid and quinacrine bulk spectra are highlighted in gray and green, respectively. Except for quinacrine powder, all spectra are presented as being collected without baseline correction.

As reported previously, gold cavity array structures create excellent SERS (surface-enhanced Raman spectroscopy) platforms.^66,74−76^ As a result, we used Raman spectroscopy of the bilayers at the array to gain molecular structural insight into the drug’s interaction with the membrane. Before SERS measurements, we first performed classical Raman measurements of the individual lipids, DOPC and cholesterol, and quinacrine drug in their powder forms on flat gold substrates. The Raman spectra of the DOPC powder control are shown in the bottom panel of Figure 5a (with characteristic features highlighted in light gray) for comparison with the band position obtained in SERS from the DOPC bilayer on MSLBs, as shown by the black lines. In all cases, the Raman shift band positions are consistent with the reported literature values.^77−81^ The main features in the Raman spectra of DOPC are observed in the fingerprint regions of the hydrocarbon chain and can be ascribed to bending, scissoring, and twisting and as well as C–C, C–H, and C=C stretching modes. For example, the bands between 1440 and 1650 cm^–1^ are associated with CH_2_ scissor bending (δ(CH_2_)) and C=C double-bond stretching (ν(C=C)), respectively. Other prominent features near 1250–1300 and 1025–1140 cm^–1^ correspond to CH_2_/CH deformation and C–C backbone single-bond stretching, respectively. Bands originating from the phospholipid head can be observed at 718, 876, 957, and 1737 cm^–1^, assigned to symmetric choline (ν_s_C–N^+^–C) stretching, antisymmetric choline (ν_as_C–N^+^–C) stretching, phosphate P–O stretching (ν(P–O)), and ester carbonyl stretching (ν(C=O)), respectively. The Raman spectrum for quinacrine powder is shown in the top panel (red, Figure 5a,b). The theoretical Raman spectrum of quinacrine (cf. Figure 1b for the energy-minimized structure) was predicted by DFT computation for the orientation-averaged spectrum by employing the CAM-B3LYP functional using the 6-311++G(d,p) basis set in the *Gaussian 09* tool, as reported previously.^82^ The experimental and theoretical data are in excellent agreement with to band assignments (Figure S3, SI). The Raman features centered at 1372, 1401, and 1469 cm^–1^ are assigned to in-plane ring-stretching vibrations and the band at 1586 cm^–1^ is assigned to the exocyclic C–N stretching mode, and these assignments are in good agreement with previously report Raman data for the drug.^83^

On the basis of this information, the experimental SERS spectra of the DOPC bilayer alone (Figure 5a, black) and in contact with quinacrine (Figure 5a, blue) were compared with the analogous spectra for the DOPC:Chol bilayer. We focused on this comparison because EIS data indicate clear evidence of interaction in the former membrane, but quinacrine interaction with the DOPC:Chol bilayer was inconclusive. The presence of quinacrine in the DOPC membrane is clearly evident from SERS spectra (blue), with intense features associated with the drug at 1361 and 1538 cm^–1^, assigned to the in-plane ring vibration and a gold-surface-bound exocyclic C–N band in its protonated form. The intensity of the drug’s Raman features indicate that it has, consistent with EIS data, successfully permeated the DOPC membrane and reached the cavity’s interior, where its Raman signature is strongly SERS-enhanced. When compared to its powder spectrum, the SERS spectra of quinacrine obtained from the DOPC MSLBs is red-shifted, which likely indicates some adsorption at the gold cavity after its permeation. The association and permeation of quinacrine across DOPC MSLBs has little impact on the headgroup region (choline), from its Raman signature. Quinacrine, on the other hand, induces a blue-shifted band of lipidic δ(CH_2_) from 1435 to 1460 cm^–1^ (Figure 5a) as well as symmetric ν_s_(CH_2_) and ν_s_(CH_3_) bands which were centered at 2850 and 2906 before the quinacrine blue shift to 2854 and 2908 cm^–1^ (Figure S4, SI). This is tentatively attributed to disordering of the DOPC alkyl chain packing, which leads to the decreased resistance observed in EIS. Figure 5b illustrates the SERS spectra of DOPC:Chol MSLBs before and after quinacrine binding, along with the powder Raman spectra of DOPC, cholesterol, and quinacrine. As expected, the SERS spectrum of DOPC:Chol is more complex than that of the pristine DOPC spectra, with the red shift of the characteristic ν_s_(P–O) band from 967 to 954 cm^–1^ evident, indicating the interaction of cholesterol with the DOPC headgroup. The ν_s_(C–N^+^–C) which was centered at 718 cm^–1^ in DOPC red shifts to 705 cm^–1^ in the cholesterol-containing bilayer. Although the quinacrine band is detectable at the DOPC:Chol membrane, the relative intensity of the drug’s Raman features is dramatically lower than that observed at the DOPC MSLB (highlighted in green, Figure 5b), indicating that the drug is not reaching the cavity’s interior.^75^ Consistent with EIS data, Raman indicates that quinacrine interaction with the membrane is restricted to the cholesterol aromatic backbone of quinacrine within the DOPC:Chol membrane, as shown by its blue shift of the δ(CH_2_) band from 1472 to 1498 cm^–1^. Quinacrine also induces further changes that may involve the membrane ordering effect of DOPC:Chol MSLBs, as reflected in νs(CH_2_) and νs(CH_3_) centered at 2846 and 2900 cm^–1^, respectively, which are red-shifted from the band observed at 2848 and 2904 cm^–1^ (Figure S4b, SI) for the pristine membrane. FLCS data further confirms the DOPC:Chol membrane’s alkyl chain ordering effect (*vide supra*).

### Fluorescence Lifetime Imaging and Lipid Diffusivity across MSLB upon Quinacrine Interaction

**Figure 6:**
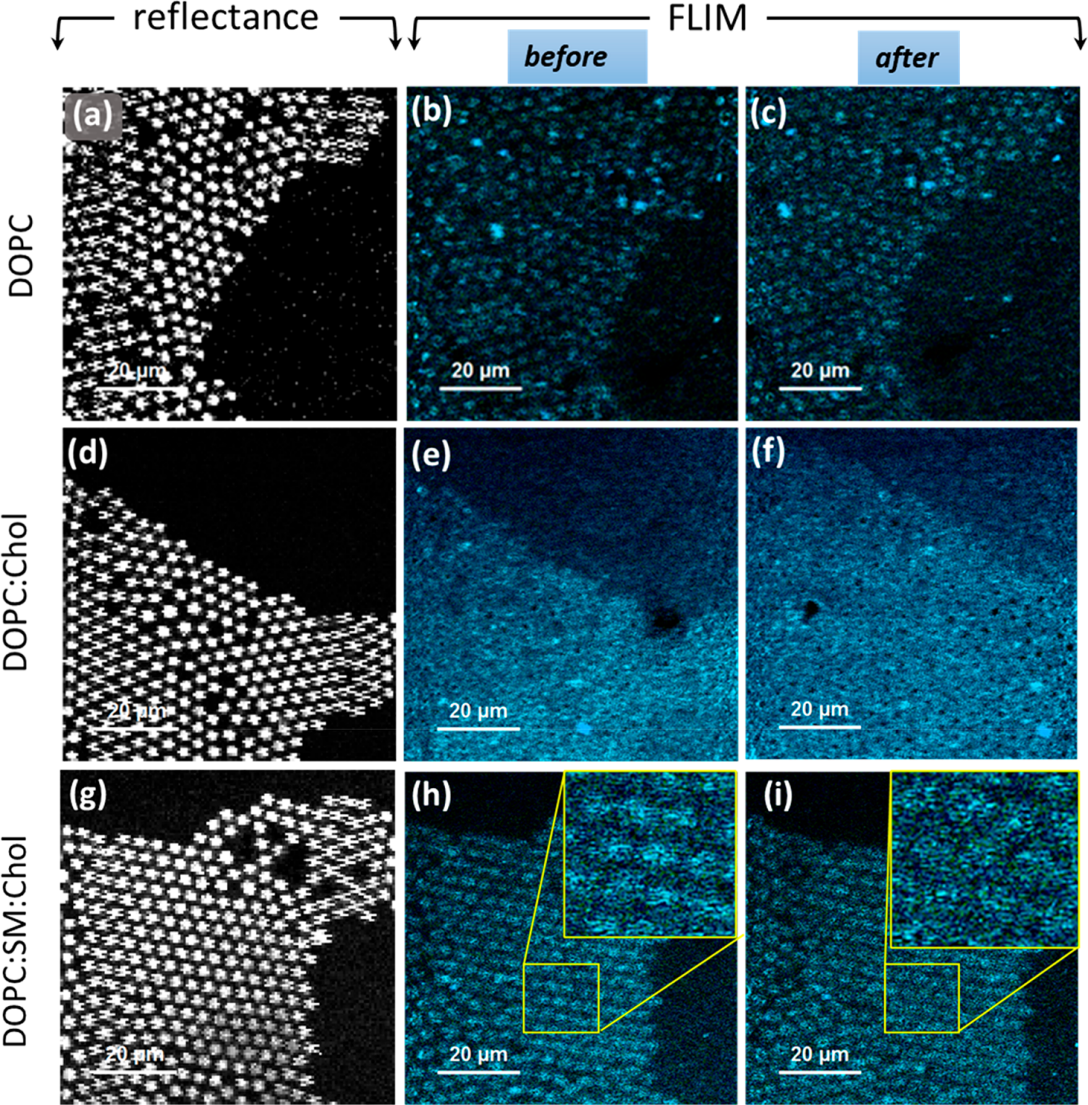
Representative reflectance and fluorescence lifetime images of MSLBs formed on a PDMS substrate. Panels a, d, and g are the reflectance images of DOPC, DOPC:Chol, and DOPC:SM:Chol MSLBs obtained from confocal microscopy, where the white circular spot represents an aqueous filled cavity and the black area corresponds to planar and/or unfilled cavities. Panels b, e, and h show the corresponding fluorescence lifetime images of the respective bilayers before drug addition. The lifetime image of bilayers is obtained from a fluorescently labeled lipid probe, DOPE-ATTO655 (0.01 mol %), present at the upper leaflet of the bilayer on the PDMS microcavity array. Panels c, f, and i show the respective lifetime images obtained after 30 min of incubation with quinacrine (∼10 μM). The scale bar in each panel is 20 μm. The insets in panels h and i are the expanded regions showing a modest membrane homogenization caused by quinacrine, highlighted by the yellow squares.

The effect of drugs on membrane packing and passive permeability is of particular interest because it has been proposed that a decrease in membrane order may affect the membrane protein activity and protein-mediated multidrug resistance.^84,85^ To gain a better understanding of these effects, we used confocal fluorescence lifetime imaging and correlation spectroscopy to probe the influence of quinacrine on the fluidity of the DOPC, DOPC:Chol, and DOPC:SM:Chol membranes in an analogous pore-suspended membrane at an optically transparent PDMS substrate. Figure 6 shows representative reflectance and fluorescence lifetime images of the three different MSLBs composed of DOPC, DOPC:Chol (3:1), and DOPC:SM:Chol (2:2:1) before and after quinacrine incubation. For FLIM and FLCS measurements, fluorescent lipid marker DOPE-ATTO655 (0.01 mol %) labels the outer leaflet of the MSLBs. Before quinacrine addition, the fluorescence marker is distributed equally over the pore array, giving homogeneous FLIM images of DOPC (Figure 6b) and DOPC:Chol (Figure 6e). In contrast, the FLIM image of DOPC:SM:Chol MSLB is heterogeneous (Figure 6h, inset), which is attributed to the expected phase separation of high-melting SM and low-melting DOPC lipid, and DOPE-ATTO655 is known to show a preference for liquid disordered (L_d_) phases.^62^

The addition of quinacrine (10 μM) had no discernible effect on the FLIM images of DOPC (Figure 6c) and DOPC:Chol (Figure 6f) membranes. In contrast, some modest membrane homogenization seems to occur after quinacrine incubation with the DOPC:SM:Chol membrane (Figure 6i, inset). Crucially, FLIM imaging confirms that the membranes remain intact throughout drug treatment in all instances, precluding membrane disruption as a contributor to the observed decrease in electrochemical resistance for DOPC and DOPC:SM:Chol (*vide infra*).

**Figure 7:**
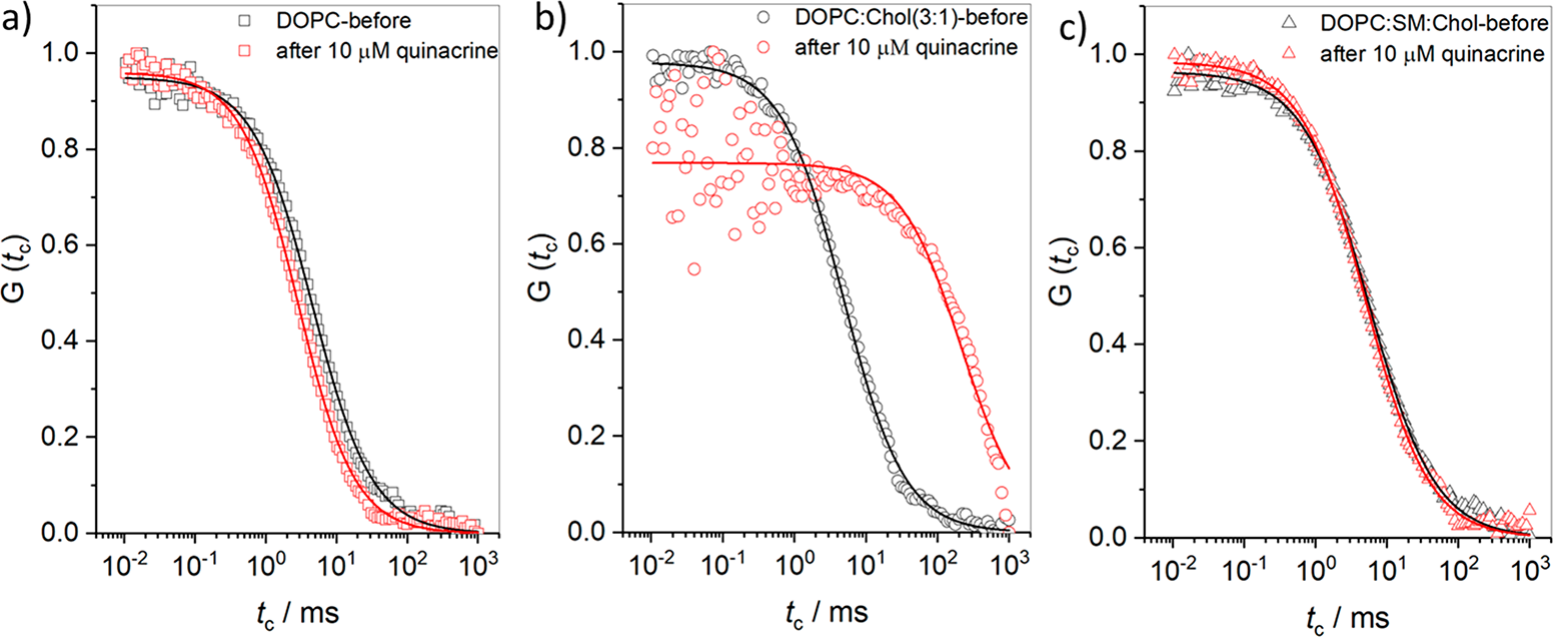
Representative FLCS autocorrelation function data obtained from different membranes such as (a) DOPC, (b) DOPC:Chol(3:1), and (c) DOPC:SM:Chol before (black open symbols) and after incubation with 10 μM quinacrine (red open symbols). The distal leaflet of all of the membranes is doped with 0.01 mol % DOPE-ATTO655. The lipid membrane spanned the ∼2 μm cavity PDMS array filled with the PBS buffer at pH 7.4. In each panel, solid lines are the representative fit using the 2D diffusion model equation. FLCS spectra were collected from the membrane at the center of pore, and data is averaged across 40–50 FLCS measurements before and after drug incubation.

Next, to evaluate the impact of quinacrine interaction on membrane order, we carried out point FLCS measurements before and after quinacrine binding at these different MSLBs. Figure 7 shows representative fluorescence lifetime autocorrelation spectroscopy data acquired from the different membrane compositions: DOPC (black, Figure 7a), DOPC:Chol (black, Figure 7b) and DOPC:SM:Chol (black, Figure 7c) prior to drug incubation at MSLBs. The 2D translational motion of the fluorescent lipid tracer, DOPE-ATTO655, is produced by FLCS collected from the pore center of a spanned bilayer (cf. Figure S6 (SI)).

The lipid diffusivity was determined by fitting the ACF data to the 2D model (eq 2), as given in the experimental methods. Before drug addition, the diffusivity values for DOPC, DOPC:Chol(3:1), and DOPC:SM:Chol were calculated to be 9.3 ± 0.6, 7.8 ± 0.3, and 6.2 ± 0.4 μm^2^/s, respectively. The data is compiled from around 40–50 measurements taken before and after drug binding for each membrane type and averaged. The trend, as expected, reflects the membrane ordering effect of cholesterol in DOPC and the DOPC:SM:Chol(2:2:1) composition; the former is expected to contain the L_d_ phase at room temperature, and the latter is expected to contain both L_o_ and L_d_ phases. When the DOPC membrane is treated with 10 μM quinacrine, the lipid diffusivity increased from 9.3 ± 0.6 to 13.03 ± 0.4 μm^2^/s (red, Figure 7a), indicating that the drug reduced the DOPC membrane packing. The result is consistent with the increased permittivity observed by EIS. Remarkably, in contrast, the lipid diffusivity of the DOPC:Chol (3:1) membrane decreased dramatically from 7.8 ± 0.3 to 0.19 ± 0.7 μm^2^/s (red, Figure 7b) on exposure to quinacrine. The anomalous parameter α, as defined in eq 1, remains at 0.99 ± 0.02 before and after drug addition, suggesting that the diffusion of DOPC and DOPC:Chol(3:1) membranes is Brownian. The magnitude of the reduced diffusivity at the DOPC:Chol membrane seems surprising considering the drug’s relatively weak impact on EIS, but it is consistent with the drug’s opposing influence on this lipid composition. It implies, along with the increased resistance of the film from EIS, that drug binding may modify the phase of the DOPC:Chol membrane, and we speculate that quinacrine reduces the phase-transition temperature such that it may form a gel phase at room temperature, drastically reducing the diffusivity. For the DOPC:SM:Chol membrane, on the other hand, consistent with the DOPC data, the drug induced a modest increase in lipid mobility from 6.2 ± 0.4 to 7.41 ± 0.25 μm^2^/s (red, Figure 7c), again associated with the reduced lateral order.

Combining the aforementioned multimodal approaches, three different pore-suspended bilayers were explored here to mimic different aspects of the phases of the eukaryotic cell membrane. Experiments using EIS and SERS indicated that quinacrine readily passively penetrates the DOPC bilayer, and data from fluorescence correlation and EIS reveals that this is accompanied by increased membrane disorder and/or thinning. These alterations are irreversible, even after buffer exchange, as shown by EIS (Figure 3a, after buffer exchange).

Cholesterol had a profound impact on permeation through the L_d_-phase membrane. When incorporated at 30% mol/mol in a DOPC bilayer, quinacrine permeation was blocked, as confirmed by SERS studies. These observations are in line with previous findings that cholesterol reduces the passive uptake of different classes of drugs^86,87^ as well as gold nanoparticles.^88^ Correspondingly, the drug had only a modest impact on membrane resistance where it was observed in contrast to DOPC alone to stimulate a small increase in resistance (Figure 4a) and a decrease in capacitance (Table 2 and Figure 4b). At higher quinacrine concentrations, drug intercalation may thicken the membrane, consistent with a prior study.^89^ From SERS, alkyl-chain-symmetric CH_2_ stretching bands indicated also that quinacrine binding impacted membrane ordering (Figure S4, SI). Small molecules may enhance the overall packing of the alkyl chain by orienting the P^–^–N^+^ dipole of the lipid headgroup.^90^ Furthermore, remarkably, the lipid diffusivity measurements by FLCS revealed lipid alkyl chain ordering (Figure 7b) with a massive decrease in membrane diffusivity evident on exposure of the binary membrane to the drug. Because DOPC:Chol (3:1) is not expected to exhibit any domains or phase separation at room temperature,^64,91^ the severe decrease in lipid diffusivity due to quinacrine binding is speculated to be due to gel-like domains within the DOPC:Chol membrane caused by the drug.

Using an empirical binding model, there is modest (*K*_a_ = 0.23 ± 0.07 L μM^–1^) affinity between quinacrine and the DOPC:Chol membrane; however, the affinity between DOPC and quinacrine is much higher (*K*_a_ = 1.27 ± 0.02 L μM^–1^). Although these values are empirical, they may explain the stated inhibitory value for lysosomal phospholipase, a mode of action suggested for antimalarial drugs.^28,92^

Compared to DOPC, DOPC:SM:Chol (2:2:1), which is a closer analogue of the eukaryotic plasma membrane, showed the largest drop in resistance and a low *K*_a_ (0.30 ± 0.01 L μM^–1^) on drug contact. The resistance values indicate that the drug increases membrane admittance possibly by reducing the packing order of SM-Chol-rich regions, resulting in ion leakage. We observed (Figure 4b) an increase in capacitance, indicating membrane thinning. At room temperature (22 ± 1 °C), a membrane composed of DOPC, SM, and Chol (2:2:1) is expected to form phase-separated domains of liquid disordered and ordered phases rich in cholesterol.^62,93^ Given that we observed greater increases in capacitance and decreases in resistance than for the DOPC (L_d_) membranes, we hypothesize that quinacrine treatment may increase the cholesterol redistribution (lipidosis) in SM-Chol rich regions, increasing the membrane disorder. Our SERS data are consistent with this. For instance, in the absence of quinacrine, the band centered at 1635 cm^–1^ (Figure S5, highlighted in red, SI) is formed by the intramolecular H-bonding interaction between SM amide I (C=O) and the cholesterol −OH group, which is red-shifted from 1643 cm^–1^ due to the SM-SM H-bond.^94^ In the presence of quinacrine, the 1642 cm^–1^ band reappears, indicating that quinacrine interacts with the SM headgroup by dislodging cholesterol from the region of SM. Our results are in line with the previous report, which found that quinacrine abolishes prion protein activity by redistributing cholesterol from the plasma membrane to intracellular membranes, thereby destabilizing the membrane domain.^95^

Reorganization of the lipid bilayer may potentially affect drug–protein and membrane–protein interactions.^96^ Quinacrine induces membrane heterogeneity (Δ*m* = −*v*e) at the DOPC:SM:Chol membrane, but not at the DOPC and DOPC:Chol membranes (Δ*m* ≈ 0), according to our EIS data (cf. Figure S7, SI), which was further corroborated using FLIM imaging. The exponent of CPE values (*m*) often defines the overall homogeneity of the membrane.^54,97^

Overall, the study shows the versatility of MSLBs as platforms for interrogating the drug–membrane interaction, though of course one disadvantage of this and other model membranes is that they do not reflect the true complexity of the plasma membrane. Lacking protein and other features such as glycocalyx, they reflect only the lipid membrane interaction and passive permeation; however, because passive permeation can be difficult to establish in cells, it is useful in this regard.

## Conclusions

We interrogated the lipid membrane interactions and permeability of multipurpose drug quinacrine at microcavity array supported lipid bilayers of different compositions. Three membrane compositions were selected to reflect membrane phases present at the cell membrane, including liquid disordered phase with and without cholesterol, and the mixed L_o_/L_d_ phases. The impact of cholesterol and phase heterogeneity induced by the mixed lipid composition on the quinacrine–membrane interaction and permeability was evaluated using electrochemical impedance spectroscopy (EIS) and surface-enhanced Raman spectroscopy (SERS) in label-free studies, and changes to the fluidity of the membrane in response to the drug was evaluated by fluorescence lifetime correlation spectroscopy.

EIS data reveals that the drug is permeable to DOPC and the ternary DOPC:SM:Chol compositions. It reduces membrane resistance and increases impedance in both cases. For the ternary membrane, the impact is greatest, indicating the possible reorganization of lipid domains as reflected by decreases in the admittance of the membrane. Conversely, permeation is arrested at the cholesterol/DOPC membrane, but the drug may bind/intercalate at the membrane interface, making it more resistant to ion permeation as reflected in non-Faradaic impedance with an increase in the admittance of the membrane. SERS confirmed membrane binding in the binary composition without permeation. While exploiting SERS to monitor the arrival of the drug at the plasmonic pore, we could unequivocally confirm membrane permeation for the primary and ternary compositions. Confocal fluorescence lifetime imaging (FLIM) confirmed that membranes remained intact during and after drug interaction, and fluorescence correlation spectroscopy (FLCS) showed that DOPC and ternary membranes undergo increased lipid diffusivity with drug binding, whereas the fluidity of the DOPC:Chol membrane decreases dramatically with drug interaction, which is speculated to be due to the lowering of the gel phase-transition temperature induced by quinacrine.

Overall, the study not only sheds light on the role of the physical properties of the membrane in the interaction with antimalarial drugs but also demonstrated that MSLB platforms holds great promise for screening the passive permeability of drugs across different biomembranes and can be extended to various other receptors such as enzymes, toxins, and protein-mediated transport across the biomembrane.

## Supporting Information Available

Pore-suspended bilayer fabrication, additional results from EIS, computational calculations, and SERS data are provided (PDF)
