# Supplementary material for: Multimodal Investigation into the Interaction of Quinacrine with Microcavity-Supported Lipid Bilayers
Source: Langmuir. 2022 May 13;38(20):6411–24. doi: 10.1021/acs.langmuir.2c00524 (PMC9134496; doi:10.1021/acs.langmuir.2c00524)
Supplement: Supplementary file 1 — la2c00524_si_001.pdf [file la2c00524_si_001.pdf]

Supporting Information

A multimodal investigation into the interaction of Quinacrine with microcavity supported lipid bilayers.

Nirod Kumar Sarangi, Amrutha Prabhakaran, and Tia E Keyes*

School of Chemical Science and National Centre for Sensor Research, Dublin City University, Dublin-9, Ireland

*Corresponding author email id: tia.keyes@dcu.ie

Table of contents:

The supporting information includes. Representative Amperometric data on gold deposition of the array and gold oxide formation for determination of surface area. AFM data on the PDMS substrate. Non-Faradaic Impedance data. Background Raman data of the Quinacrine powder, and alkyl CH stretch spectra from the cavity supported lipid bilayers. Confocal imaging of the cavity supported lipid bilayer and membrane homogeneity (m) plot.

Number of pages: 6 (page S1-page S6)

Number of figures: 7 (Figures S1-S7)

References for SI reference citations

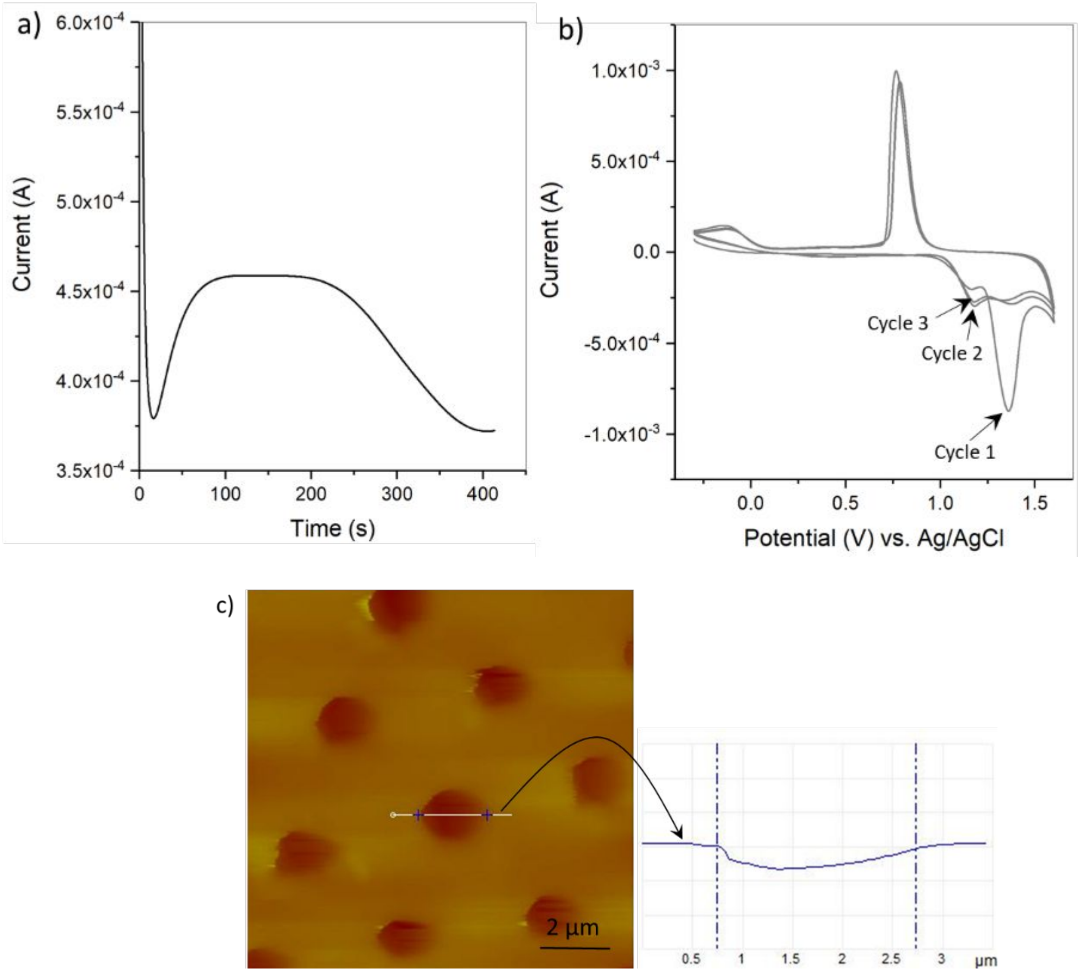

Figure S1. a) Amperometric I-t curve obtained during gold deposition at -0.6V (vs. Ag/AgCl(1M KCl)) on to the PS covered gold substrate. b) A representative cyclic voltammogram (3 cycles) obtained from 0.05M H_2SO_4. In each panel gold substrate was used as working, Ag/AgCl (1 M KCl) as reference and platinum coiled electrode as counter electrode. c) AFM topography image of PDMS cavity array shows a hexagonally packed cavity array with an individual pore diameter of ~ 2 µm (see curved arrow). The image was taken under liquid with a scan rate of 0.3 Hz using Veeco Bioscope II system (Nanotec House, Cambridge) coupled with Zeiss Axiovert inverted optical microscope IX70 with silicon nitride cantilevers PNP-TR-20 (NANO WORLD).

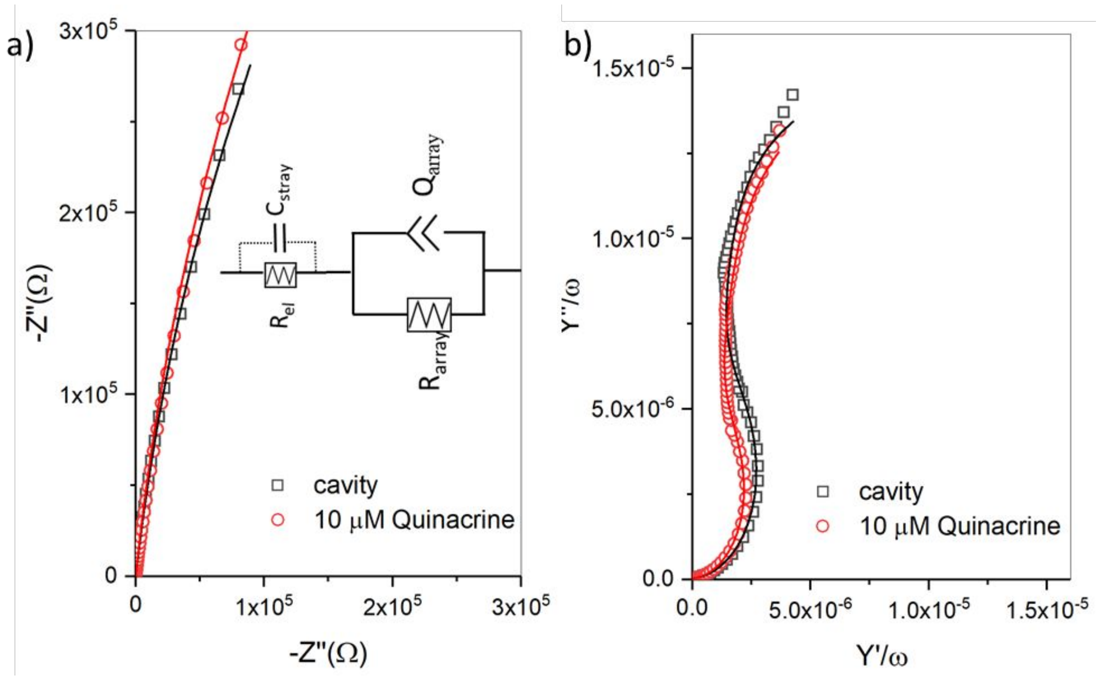

Figure S2. Non-Faradaic a) Nyquist and b) frequency normalized complex capacitance plot of SAM modified cavity array before (black square) and after 10 μM Quinacrine (red). In each panel, solid lines are the fit using ECM model shown in inset Fig.S2a. EIS measurements are carried out at 0.01 M PBS buffer (pH=7.4) at 22±1 °C using gold cavity array working, Ag/AgCl(1M KCl), reference and Pt coiled as counter electrode.

Computational methods:

Density functional theory (DFT) level energy minimization and frequency calculations for quinacrine drug were performed using CAM-B3LYP in the 6-311++G(d,p) basis set in the Gaussian 9 computational package.[1] The CAM-B3LYP functional was selected for comparison against experimental bulk Raman spectra of quinacrine in powder form because of its long-range corrected behaviour. Optimization was performed on the neutral quinacrine followed by frequency analysis using GaussSum 3.0.[2] An excitation of 785 nm similar to experimental spectra acquisition and temperature was 289 K was used for the generation of Raman spectrum. The theoretical spectrum was broadened to Lorentzian lineshapes with fwhm (full-width half-maximum) of 20 cm^{-1}.

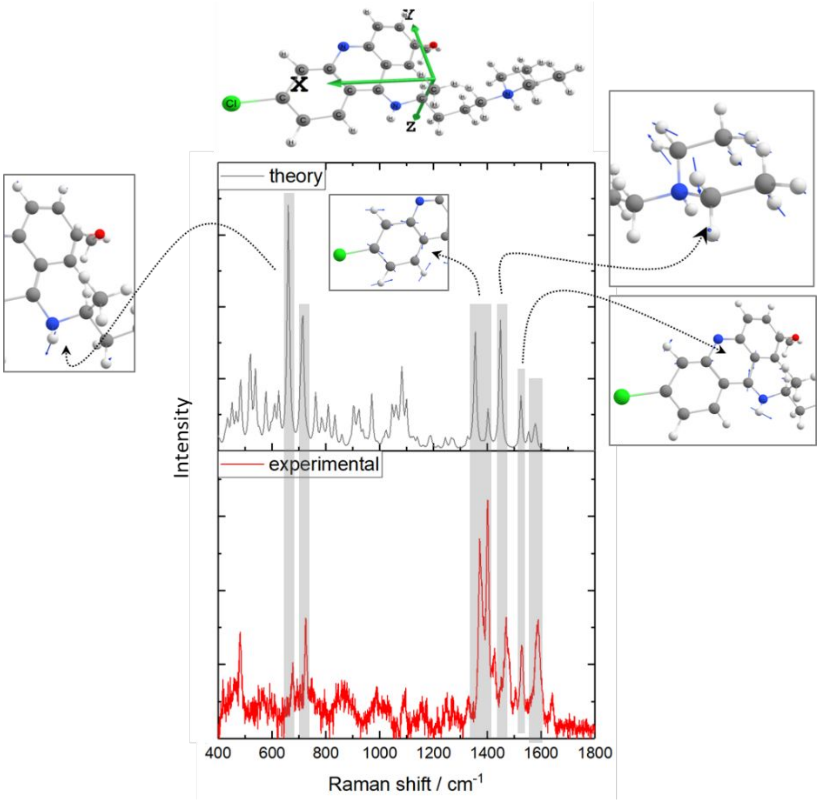

Figure S3. Illustrates experimental Raman spectrum obtained from quinacrine powder cast onto a flat gold substrate (bottom, red) and the theoretical DFT(CAM-B3LYP) Raman spectrum of neutral quinacrine (top panel, grey). The bands responsible due to the polarization effect of the corresponding functional bond along with their main molecular axis (xyz) are shown by the curved arrow next to the theoretical spectra.

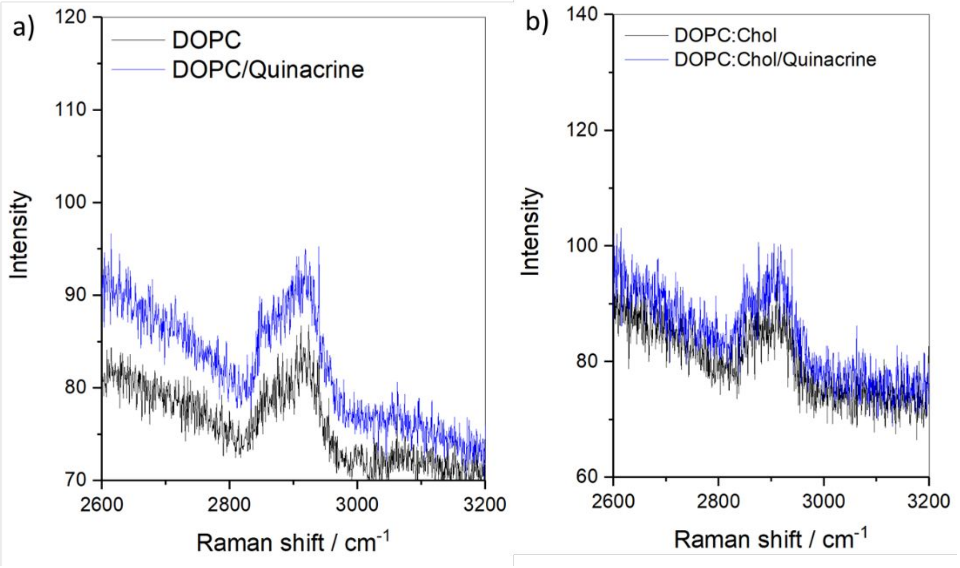

Figure S4. SERS spectra in the region of alkyl C-H stretching regions obtained from a) DOPC and b) DOPC:Chol(3:1) MSLB before (black) and after (blue) quinacrine (10 μM) binding.

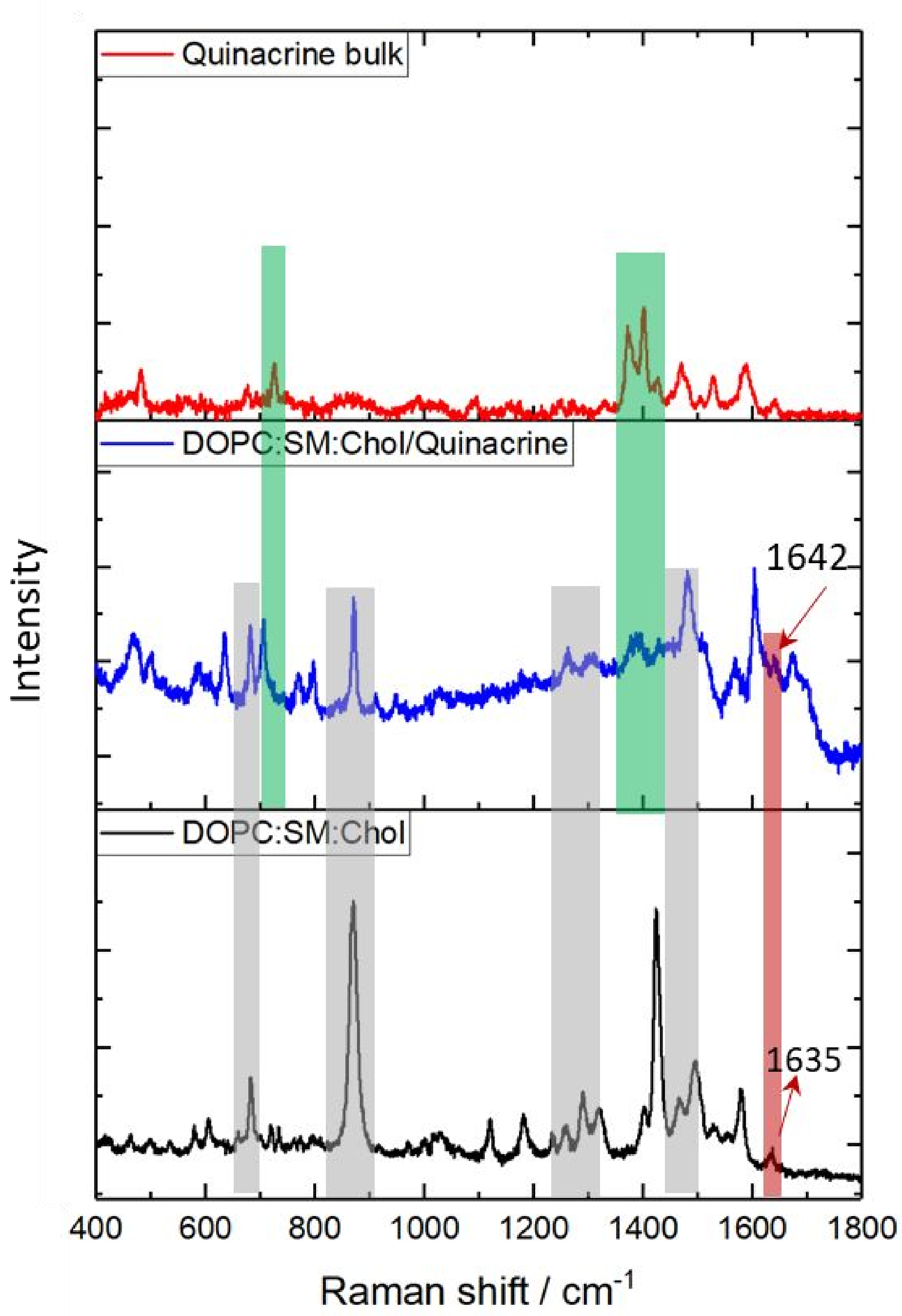

Figure S5. SERS spectra of DOPC:SM:Chol (1:1:0.5) MSLB before (black line) and after (blue line) quinacrine interaction. The bulk spectrum of quinacrine is shown in top panel (red line).

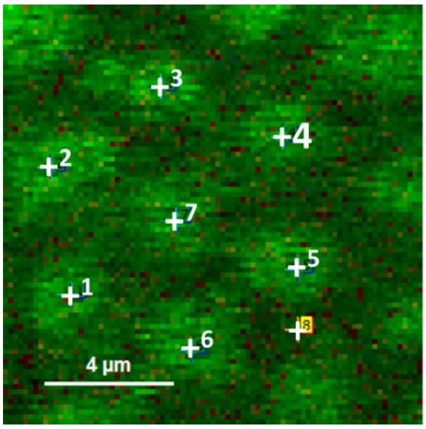

Figure S6. A representative zoomed-in image of DOPC MSLB over PDMS indicating the spatial point (numbered 1-8 marked in '+') where FLCS measurements are taken. Point number 1-7 are from the center of bilayer spanned cavity and number 8 are from the flat regime.

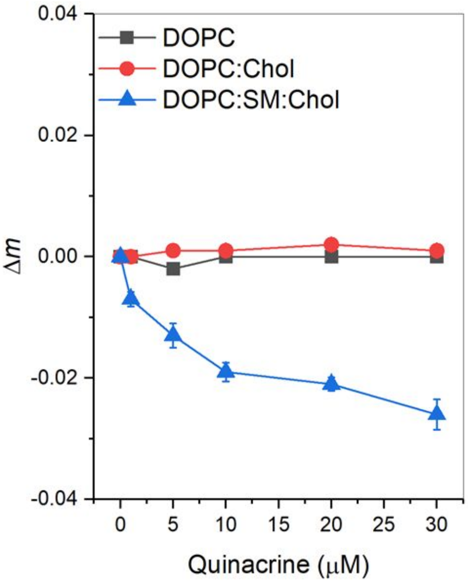

Figure S7. Relative change (Δ) in membrane homogeneity (m) values extracted from ECM fit as CPE_M exponent for different lipid compositions with quinacrine titration. The error bars are standard deviations extracted from experiments performed in triplicate for each bilayer type.

(1) Frisch, M. J et al. Gaussian 09, Revision A.02, Gaussian, Inc., Wallingford CT, **2016**.

(2) O'boyle, N. M.; Tenderholt, A. L.; Langner, K. M. Cclib: A Library for Package-Independent Computational Chemistry Algorithms. *J. Comput. Chem.* **2008**, *29* (5), 839–845.
